# Ultrasensitive Electrochemical Sensor Based on Polyelectrolyte Composite Film Decorated Glassy Carbon Electrode for Detection of Nitrite in Curing Food at Sub-Micromolar Level

**DOI:** 10.3390/molecules23102580

**Published:** 2018-10-09

**Authors:** Jingheng Ning, Xin Luo, Min Wang, Jiaojiao Li, Donglin Liu, Hou Rong, Donger Chen, Jianhui Wang

**Affiliations:** School of Chemistry and Biological Engineering, Changsha University of Science & Technology, Changsha 410110, China; jinghengning@csust.edu.cn (J.N.); luoxin_gl@126.com (X.L.); wang_min1993@126.com (M.W.); lijiaojiao_2013@126.com (J.L.); dong993@163.com (D.L.); hourong0406@163.com (H.R.); chendonger9@163.com (D.C.)

**Keywords:** polyelectrolyte composite film, nitrite detection, differential pulse voltammetry, cyclic voltammetry

## Abstract

To ensure food quality and safety, developing cost-effective, rapid and precision analytical techniques for quantitative detection of nitrite is highly desirable. Herein, a novel electrochemical sensor based on the sodium cellulose sulfate/poly (dimethyl diallyl ammonium chloride) (NaCS/PDMDAAC) composite film modified glass carbon electrode (NaCS/PDMDAAC/GCE) was proposed toward the detection of nitrite at sub-micromolar level, aiming to make full use of the inherent properties of individual component (biocompatible, low cost, good electrical conductivity for PDMDAAC; non-toxic, abundant raw materials, good film forming ability for NaCS) and synergistic enhancement effect. The NaCS/PDMDAAC/GCE was fabricated by a simple drop-casting method. Electrochemical behaviors of nitrite at NaCS/PDMDAAC/GCE were investigated by cyclic voltammetry (CV) and differential pulse voltammetry (DPV). Under optimum conditions, the NaCS/PDMDAAC/GCE exhibits a wide linear response region of 4.0 × 10^−8^ mol·L^−1^~1.5 × 10^−4^ mol·L^−1^ and a low detection 1imit of 43 nmol·L^−1^. The NaCS/PDMDAAC shows a synergetic enhancement effect toward the oxidation of nitrite, and the sensing performance is much better than the previous reports. Moreover, the NaCS/PDMDAAC also shows good stability and reproducibility. The NaCS/PDMDAAC/GCE was successfully applied to the determination of nitrite in ham sausage with satisfactory results.

## 1. Introduction

Nitrite is widely used as a preservative in food industry, especially in the production and processing of cured meat and fishery products [1]. Additionally, it is also utilized as a fertilizing agent in agriculture and an inhibitor in corrosion science [2]. Furthermore, nitrite has become one of the widespread inorganic contaminants present in soil, food, ground water, and even physiological systems [3]. Nitrite has been proven to be of potential risk to human health and leads to many serious diseases, such as methemoglobinemia, esophageal cancer, spontaneous abortion, and birth defects of the central nervous system [4,5,6,7]. Therefore, the content of nitrite demands strict control. The World Health Organization (WHO) has set a fixed maximum limit in drinking water for nitrite as 65 μM [8]. In China, according to national standard GB 2760-2014, the maximum permitted level of nitrite in meat food is 0.15 g/kg [9]. To ensure food quality and safety, developing cost-effective, rapid, and precision analytical techniques for quantitative detection of nitrite is highly desirable.

To date, many techniques have been developed for the detection of nitrite, including chromatography [10,11], UV spectrophotometry [12,13], fluorometry [14,15], and flow injection analysis [16,17]. Although these analytical methods are well-recognized, they also suffered from limits, such as high cost, being time-consuming, cumbersome, and requiring complicated pretreatment. Recently, electrochemical analysis has become one of most preferred techniques to detection small biomolecules, food additives, and environmental contaminants, owing to its overwhelming merits including cost- and time-effective, facile operation, and high sensitivity and specificity [18,19,20,21,22,23]. In the last few years, electrochemical nitrite sensors have received increasing attention. Various modified materials have been proposed for nitrite sensors, including graphene [24,25], carbon nanotubes [26], metal or metal oxide nanoparticles [27,28,29], metal organic frameworks [30,31], and conducting polymers [32,33]. However, these modified electrodes have several drawbacks, such as low detection limits (almost at the mmol/L level, far lower than sub-micromolar level for HPLC and fluorometry), narrow linear range, and susceptibility to interference. Thus, it is urgent to develop novel electrochemical sensors for the detection of nitrite at sub-micromolar level.

Poly (dimethyldiallylammonium chloride) (PDMDAAC) is a ubiquitous linear cationic polymer that has unique properties, such as biocompatibility, low cost, high charge density, as well as good water solubility [34]. It has been reported that PDMDAAC film was successfully immobilized on electrochemical sensors [35,36,37]. These previous studies demonstrated that PDMDAAC film has excellent conductivity due to the presence of electron conductive networks, and good ion-exchange performances that can effectively increase the concentration of anions round the film as compared with the bulk solution. For these reasons, PDMDAAC film was chosen as a modified material to construct electrochemical sensors, aiming to facilitate preconcentration and enhance selectivity properties of nitrite sensors, and eventually enhance the electrochemical response. However, PDMDAAC was found to exhibit relatively poor performances in film formation in our preliminary experiments, which could influence the electrode stability. Additionally, the sensitivity of PDMDAAC modified electrode is very limited. To overcome these problems, PDMDAAC was combined with sodium cellulose sulfate (NaCS), which has good film-formation ability. NaCS/PDMDAAC is an emerging composite film that has unique properties such as excellent mechanical strength, transparency and small pore size [38]. NaCS/PDMDAAC has been widely used in the immobilization of bacteria, fungus and microalgae, drug delivery systems, and membrane systems [38,39,40]. However, to our best knowledge, NaCS/PDMDAAC has rarely been applied as a modified material in electrochemical sensors. 

**Scheme 1:**
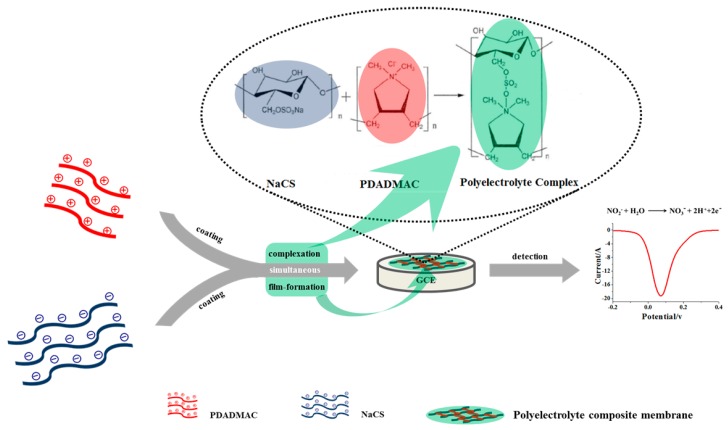
Schematic of NaCS/PDMDAAC/GCE for the detection of nitrite detection.

Herein, NaCS/PDMDAAC composite film as prepared to construct a novel electrochemical sensor for the detection of nitrite at the sub-micromolar level, aiming to make full use of the merits of individual components (biocompatible, low cost, good electrical conductivity for PDMDAAC; non-toxic, abundant raw materials, and the good film forming ability of NaCS) and synergistic enhancement effect (Scheme 1). NaCS/PDMDAAC/GCE was fabricated by coating the PDMDAAC and NaCS sequentially on the surface of GCE. The electrochemical performance were investigated by cyclic voltammetry (CV) and electrochemical impedance spectroscopy (EIS) using [Fe(CN)_6_]^3−/4−^ as redox probe solution. The analytical parameters (including pH) were systemically explored. Finally, the content of nitrite in ham sausage was determined by differential pulse voltammetry (DPV) with satisfactory results.

## 2. Results and Discussion

### 2.1. Characterization of Surface Morphology

**Figure 1:**
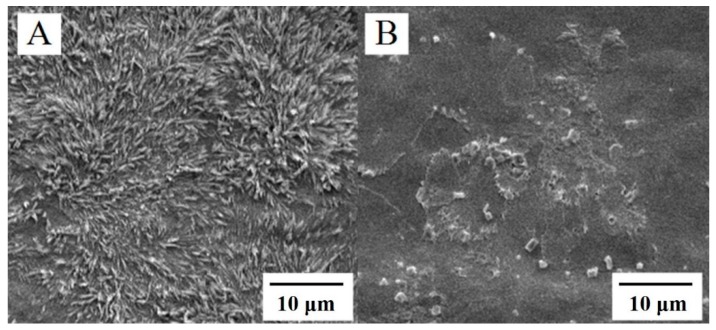
SEM images of NaCS film (**A**) and NaCS/PDMDAAC composite film (**B**).

**Figure 2:**
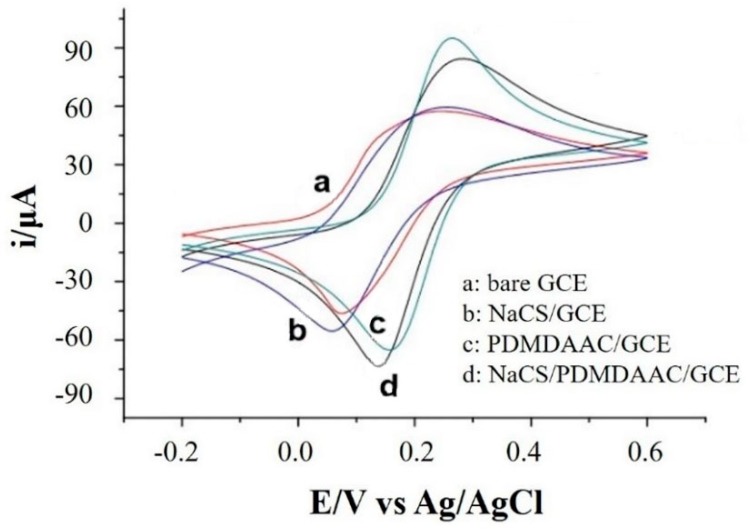
Cyclic voltammograms of bare GCE (a), NaCS/GCE (b), PDMDAAC/GCE (c), and NaCS/PDMDAAC/GCE (d) recorded in 5 × 10^–3^ mol·L^−1^ K_3_[Fe(CN)_6_] solution.

The surface morphologies of NaCS film and NaCS/PDMDAAC composite film were characterized by scanning electron microscope (SEM, Figure 1). As shown in Figure 1A, the NaCS film consists of acicular crystal structure. After compositing with NaCS/PDMDAAC, the surface is changed from acicular crystal to smooth film (Figure 2a), which is favorable for the electron transfer between the surface of modified electrode and analytes, and would eventually enhance electrocatalytic activity toward nitrite.

### 2.2. Electrochemical Characterization of NaCS/PDMDAAC/GCE

The electrochemical performance of bare GCE, NaCS/GCE, PDMDAAC/GCE, and NaCS/PDMDAAC/GCE were investigated using cyclic voltammetry (CV) recorded in 5 × 10^−3^ mol L^−1^ K_3_[Fe(CN)_6_] solution. The cyclic voltamograms recorded on various electrodes are shown in Figure 2. A pair of redox peaks appears on all the electrodes, suggesting the electrochemical process is quasi-reversible. A pair of weak and broad peaks was observed on the bare GCE (curve a), with redox peak separation (∆*E*_p_) of 137 mV. The anodic peak current (*i*_pa_) and cathodic peak current (*i*_pc_) is 43 μA, and 58 μA, respectively. On the NaCS modified electrode (curve b), the ∆*E*_p_ increases to 186 mV. Meanwhile, the peak currents also increase slightly, with *i*_pa_ of μA and *i*_pc_ of 69 μA, respectively. When modified with PDMDAAC alone (curve c), a pair of obvious and well-shaped redox peaks appears with a significant decrease on ∆*E*_p_ (101 mV). The corresponding redox peaks increase greatly, with *i*_pa_ of 81 μA and *i*_pc_ of 80 μA, respectively. When modified with NaCS/PDMDAAC composite film (curve d), the ∆*E*_p_ (143 mV) has a slight increase compared to that on the NaCS/GCE. The *i*_pa_ and *i*_pc_ is 72 μA and 89 μA, indicating that the NaCS/PDMDAAC film facilitates the electron transfer between the electrode and analyses, and eventually improves the electrochemical response. 

**Figure 3:**
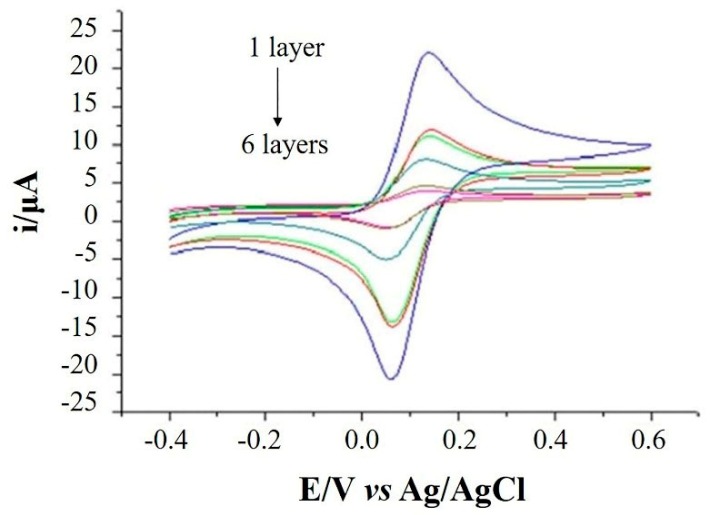
Cyclic voltammograms of different layer of PDMDAAC/NaCS modified GCE recorded in 5 × 10^–3^ mol·L^–1^ K_3_[Fe(CN)_6_] solution.

The influence of layers of NaCS/PDMDAAC composite film was also investigated in 5 × 10^−3^ mol L^−1^ K_3_[Fe(CN)_6_] solution by CV. As illustrated in Figure 3, the response peak currents decrease with the increase of layers of NaCS/PDMDAAC composite film. It is probably related to the charge transfer resistance that increases with the thickness increasing. Hence, monolayer NaCS/PDMDAAC composite film was employed for subsequent experiments.

**Figure 4:**
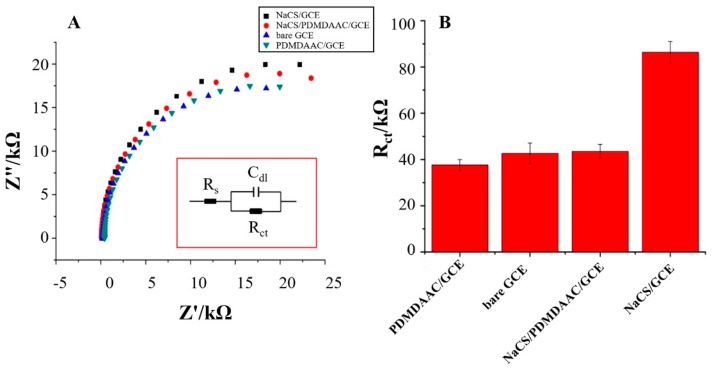
(**A**) Nyquist plots of different electrodes in 5 × 10^–3^ mol·L^–1^ K_3_[Fe(CN)_6_] solution obtained from electrochemical impedance spectroscopy (EIS). The frequency investigated was between 10,000 and 0.1 Hz with a pulse amplitude of 5 mV. The inset is the equivalent circuit of electrode/electrolyte interface. (**B**) The R_ct_ of different electrodes by EIS fitting.

Electrochemical impedance spectroscopy (EIS) is a useful tool to acquire abundant information about electrode interface, which have been widely used in the electrocatalysis [41,42], chemosensors, and biosensors [43,44]. The EIS of bare GCE, NaCS/GCE, PDMDAAC/GCE, and NaCS/PDMDAAC/GCE were also measured in the 5 × 10^–3^ mol L^–1^ K_3_[Fe(CN)_6_] solution. The Nyquist plots of different electrodes were shown in Figure 4. The charge transfer resistance (R_ct_) were estimated by EIS fitting with the equivalent circuit. The R_ct_ of bare GCE, NaCS/GCE, PDMDAAC/GCE, and NaCS/PDMDAAC/GCE are 37,830 Ω, 104,800 Ω, 36,810 Ω, and 52,190 Ω, respectively, i.e., the conductivity (1/R_ct_) of them are 2.6434 × 10^–5^ S, 9.5420 × 10^–6^ S, 2.7167 × 10^–5^ S, and 1.9161 × 10^–5^ S, respectively. Apparently, the conductivity of the bare GCE (2.6434 × 10^–5^ S) is slightly lower than that of PDMDAAC/GCE (2.7167 × 10^–5^ S) but much higher than that of NaCS/GCE (9.5420 × 10^–6^ S), indicating that when each of them is independently modified onto the bare electrode, the polycation PDMDAAC can improve the conductivity while the polyanion NaCS is not favorable for electron transfer. As is well known, both PDMDAAC and NaCS are polyelectrolytes and, theoretically, should have good conductivities. However, these polyelectrolytes are quite different in charge density, solubility, and counter-ion effects in solution (ions will naturally attract the opposite-charged ions to form counter-ion layers), all of which are thought to be able to influence the conductivity, and the former two factors are thought to be favorable for conductivity while the last one is unfavorable. In fact, the molecular weight of PDMDAAC used in our experiment is less than 1.0 × 10^5^, while NaCS has a much bigger average molecular weight more than 1.9 × 10^6^, and so it is obvious that PDMDAAC contains much shorter molecular chains, which reinvests it with a higher charge density, a better solubility, as well as a weaker counter-ion effect due to narrower interfaces to attract less opposite-charged ions. Thus, although both being polyelectrolytes, PDMDAAC can improve the conductivity of the modified GCE while NaCS cannot, as the above results show. However, it is worth noting that when using the composite NaCS/PDMDAAC modifies the bare GCE, the R_ct_ of NaCS/PDMDAAC/GCE (52,190 Ω) is only about half of that on the NaCS/GCE (104,800 Ω), suggesting the unfavorable counter-ion effect of NaCS is significantly weakened after the introduction of PDMDAAC, which actually improves the charge transfer efficiency. Obviously, the conductivity of NaCS/PDMDAAC/GCE (1.9161 × 10^−5^ S) is better than that of NaCS/GCE (9.5420 × 10^–6^ S). Thus, by considering the outstanding conductivity of PDMDAAC as well as the good performances of NaCS for film formation, the composite NaCS/PDMDAAC is the most suitable material for the modification of GCE to construct the new and effective sensor for nitrite detection, herein with a moderate R_ct_/conductivity value, which is consistent with the above research result conducted by the CV method. 

### 2.3. Electrochemical Behavior of Nitrite on NaCS/PDMDAAC/GCE

**Figure 5:**
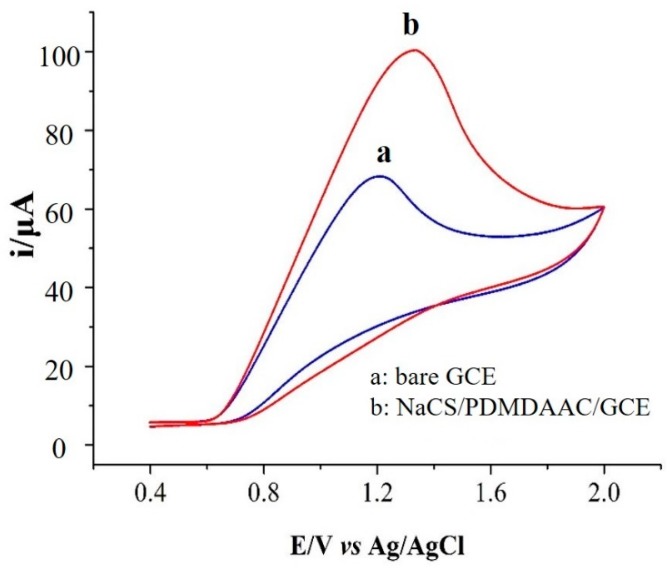
Cyclic voltammograms of bare GCE (a) and NaCS/PDMDAAC/GCE (b) in 0.1 mol·L^–1^ PBS solution (pH 7.0) containing 4.0 × 10^–3^ mol·L^–1^ nitrite.

The electrochemical behaviors of nitrite on bare GCE and NaCS/PDMDAAC/GCE were investigated by CV in 0.1 mol·L^–1^ PBS solution containing 4.0 × 10^–3^ mol·L^–1^ nitrite (Figure 5). The anodic peak current on the bare GCE is 59 μA. When modified with NaCS/PDMDAAC composite film, the anodic peak current increases to 97 μA, suggesting the NaCS/PDMDAAC composite film improve the electrochemical responses of nitrite.

### 2.4. Optimization of Analytical Conditions

#### 2.4.1. Effect of the Modifier Loading Amount

**Figure 6:**
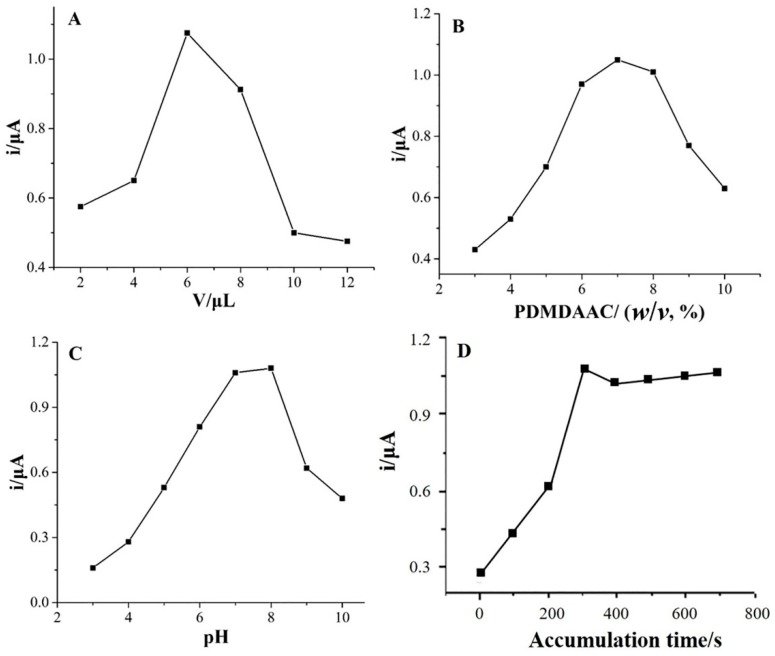
Effect of loading amount of NaCS/PDMDAAC (**A**), concentration of PDMDAAC(*w*/*v*) (**B**), pH (**C**) and accumulation time (**D**) on the response peak current of nitrite (1.0 × 10^–4^ mol·L^–1^) at the NaCS/PDMDAAC/GCE.

The effect of the modifier loading amount is one of important factors that affect the electrochemical response of modified electrode. The bare GCEs were modified with various dosage of NaCS/PDMDAAC dispersion (*v*/*v* = 1:1) firstly. Then the response peak currents of 1.0 × 10^–4^ mol·L^–1^ nitrite on these electrodes were recorded and compared. The effect of modifier loading on the response peak current was shown in Figure 6A. The response peak currents of nitrite vary with the loading amount of NaCS/PDMDAAC composite. The response peak current increases gradually with the loading amount increasing from 2–6 μL, afterwards the response peak currents decrease with the further increase on loading amount. The maximum response peak current is obtained when the loading amount is 6 μL. Namely, the loading amounts of PDMDAAC and NaCS are 3 μL. Hence, the 6 μL loading amount was used in the subsequent experiments. The trend of response peak current to loading amount is directly related to the amount of reactive active sites and the thickness of modifier films. With loading amount increasing, the amount of available reactive active sites increases, which causes the increase on the response peak current. However, the thickness of the modified film increases when the loading amount beyond 6 μL, which hinders the electron transfer between the electrode and nitrite. As a result, the response peak current decreases with the further increase on the loading amount.

#### 2.4.2. Effect of PDMDAAC Concentration

The effect of the PDMDAAC concentration on the response peak current of nitrite was also explored. The results are shown in Figure 6B. It observed that the response peak current increases as the increase of PDMDAAC concentration. When the concentration (*w*/*v*) is 7%, the peak current reaches the maximum. Afterwards the response peak current decreases with further increase on PDMDAAC concentration. Therefore, 7% was selected to be the suitable PDMDAAC concentration for the following measurements.

#### 2.4.3. Effect of pH

It is well known that pH plays a crucial role on the oxidation of nitrite, so it’s well worth investigating the influence of pH. The response peak currents of 1.0 × 10^–4^ mol·L^–1^ nitrite were measured in various pH (3.0–10.0), and the result was presented in Figure 6C. When the electrolyte solution closes to neutral (pH 7.0 or 8.0), the response peak current is largest. Therefore, pH 7.0 was recommended as the optimum pH. Under acidic conditions, NO_2_^-^ is very unstable and easily reacted with H^+^ to produce NO and NO_2_ gas. As a result, the response peak current declines as the pH decreasing. In addition, most amount of the NO_2_^−^ is protonated in such acidic environment (pK_a_(HNO_2_) = 3.3). Under alkaline pH ranging of 8.0–10.0, the increase on pH leads to the decline of the response peak current due to the lack of sufficient H^+^. 

#### 2.4.4. Effect of Accumulation Time

As we all know, accumulation time directly influences the adsorption of analyses at the surface of modified electrode. The NaCS/PDMDDA/GCE was accumulated for various times firstly, then corresponding response peak currents were record and compared (Figure 6D). During the first 300 s, the response peak currents increase with the time prolonging. It is probably due to the increasing amount of nitrite adsorbed on the reaction active sites. The highest response peak current was obtained for 300 s accumulation. Afterwards the response peak currents remain stable, suggesting the nitrite adsorption has achieved saturation. Thus, 300 s was selected as the optimal accumulation time.

### 2.5. Standard Curves, Linear Ranges, and Detection Limit

**Figure 7:**
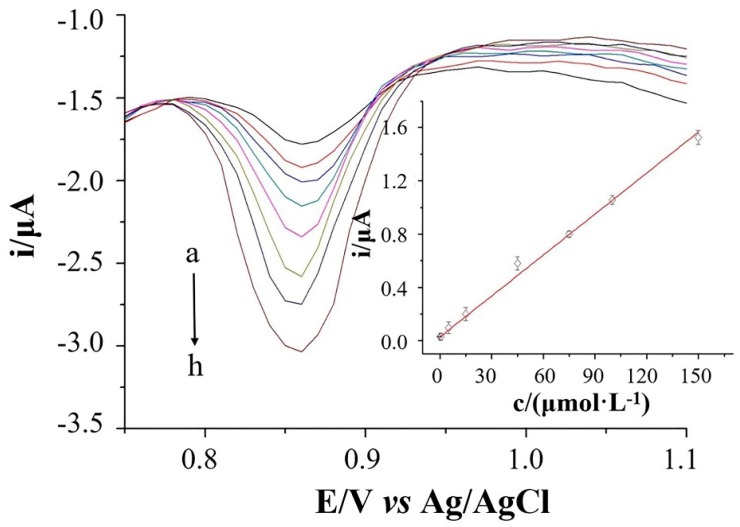
Differential pulse voltammograms of NaCS/PDMDAAC/GCE in the nitrite solution of different concentrations with 0.1 V pulse amplitude, 0.01 s pulse width and 0.1 V s^−1^ scan rate. (a) 1.5 × 10^−4^ mol·L^–1^; (b) 1.0 × 10^−4^ mol·L^–1^; (c) 7.5 × 10^−5^ mol·L^–1^; (d) 4.5 × 10^−5^ mol·L^–1^; (e) 1.5 × 10^−5^ mol·L^–1^; (f) 5 × 10^−6^ mol·L^–1^; (g) 5 × 10^−7^ mol·L^–1^; (h) 4 × 10^−8^ mol·L^–1^.

Under the optimized analytical conditions, the response peak currents of various concentrations of nitrite were measured by NaCS/PDMDAAC/GCE using differential pulse voltammetry (DPV). The response DPV curves of various concentrations of nitrite are shown in Figure 7. There is a good relationship between the response peak current and the nitrite concentration ranging from 4.0 × 10^−8^ mol·L^–1^ to 1.5 × 10^–4^ mol·L^–1^. The corresponding linear regression equation can expressed as *i* (μA) = 0.01027*c* (μmol·L^–1^) + 0.02771 (*r* = 0.9984, *n* = 8). The limit of detection (LOD, S/N = 3) is estimated to 4.3 × 10^–8^ mol·L^–1^. A comparison on sensing performances between reported electrodes and NaCS/PDMDAAC/GCE is summarized on the Table 1. The sensing performances (in terms of linear response range and limit of detection) of the NaCS/PDMDAAC/GCE are at least comparable to, and even better than, the most reported modified electrodes [45,46,47,48,49,50].

### 2.6. Selectivity, Reproducibility, and Stability of NaCS/PDMDAAC/GCE

Before detection nitrite in real samples, the practicability (including selectivity, reproducibility, and stability) of the proposed NaCS/PDMDAAC/GCE were checked carefully. Firstly, the interference investigation was performed in 1 × 10^–5^ mol·L^–1^ sodium nitrite mixed with the equal concentration of potential interfering substances including sodium chloride, glucose, l-cysteine, and ascorbic acid. The response peak current is hardly changed with these interfering compounds (peak current variation within 5%), indicating the nitrite is bonded specifically to the NaCS/PDMDAAC composite film and interfering substances cannot interfere with the nitrite detection. In other words, the proposed sensor has good specificity and selectivity toward nitrite. 

Then, the reproducibility of NaCS/PDMDAAC/GCE was validated by repeatedly measuring the response peak currents for 9 times in 8.0 × 10^−5^ mol·L^‒1^ and 8.0 × 10^‒6^ mol·L^–1^ nitrite solution alternately. The result is shown in Table 1. Obviously, the response peak current remains stable after repeated measurements for 9 times, with relative standard deviation (RSD) of 1.05% and 4.89%, respectively (Table 2). It demonstrates that the proposed NaCS/PDMDAAC exhibits good reproducibility for nitrite detection.

**Figure 8:**
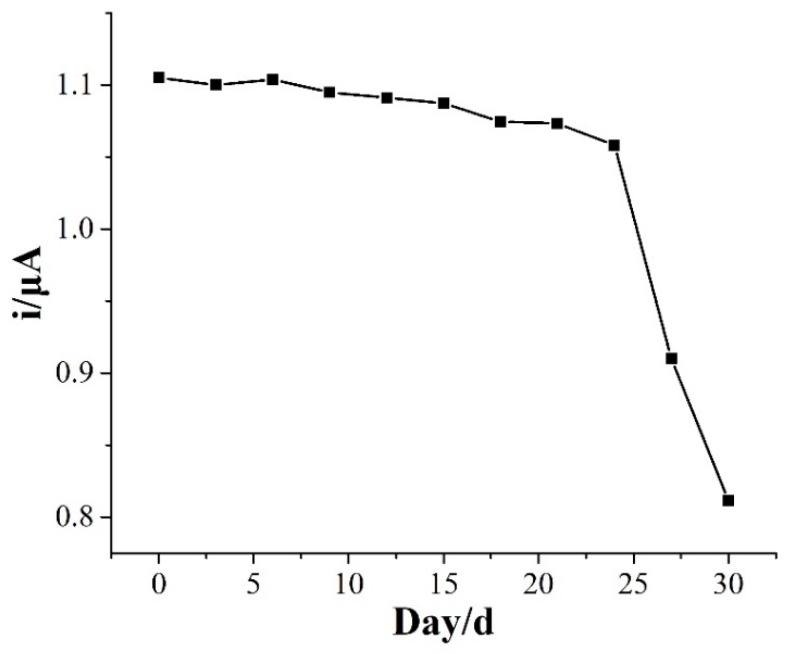
Stability for NaCS/PDMDAAC/GCE monitored in the 1.0 × 10^−4^ mol·L^–1^ nitrite for 30 days.

Finally, the stability of the proposed sensor was investigated by continuously monitoring the response peak current for 30 days. The NaCS/PDMDAAC/GCE was always immersed in 0.1 mol·L^‒1^ PBS solution (pH 7.0) and stored at 4 °C for 30 days. The cyclic voltammetric response of nitrite at the NaCS/PDMDAAC/GCE was measured every 3 days. As shown in Figure 8, after 24 days, the current response of nitrite remains unchanged nearly. After 30 days, the response peak current value still retains 73.6% of the initial value. It can be inferred that the NaCS/PDMDAAC/GCE have good stability, which is very especially important for real sample detection.

### 2.7. Determination of Actual Samples

Under the optimal analytical conditions, the pretreated ham sausage filtrate was used as the actual sample. The concentration of nitrite was detected by DPV method in 0.1 mol·L^–1^ PBS (pH 7.0) using the proposed NaCS/PDMDAAC/GCE, and determination results are listed in Table 3. The unspiked content of nitrite are 26.24−27.32 mg/kg, which does not exceed the maximum permitted content (0.15 g/kg) [9]. Then these samples were spiked with known concentration nitrite, the recovery is estimated to 98.2–104.0%, with relative standard deviation (RSD) lower than 3.3%, suggesting the satisfactory results are obtained. Together with outstanding advantages including low cost, sensitivity, and selectivity, the proposed NaCS/PDMDAAC/GCE shows great prospects on the detection of nitrite in diverse food samples.

## 3. Conclusions

In this work, a novel electrochemical sensor based on NaCS/PDMDAAC composite film was proposed for the detection of nitrite at sub-micromolar level. The NaCS/PDMDAAC/GCE was fabricated by a facile drop-casting method. The surface morphology of NaCS film and NaCS/PDMDAAC composite was characterized by SEM. The electrochemical behavior of nitrite at the NaCS/PDMDAAC was investigated by CV and DPV. The NaCS/PDMDAAC not only shows the inherent properties from the individual component, but also exhibits synergetic enchantment effect toward sensing nitrite. Under optimal detection conditions, the proposed NaCS/PDMDAAC/GCE exhibits a wide linear response region of 4.0 × 10^−8^ mol·L^–1^~1.5 × 10^−4^ mol·L^−1^, and low detection limit of 43 nmol·L^–1^ (S/N = 3). Moreover, the proposed NaCS/PDMDAAC/GCE also has good stability and reproducibility. Finally, the NaCS/PDMDAAC/GCE was successfully used to detect the nitrite content in the ham sausage sample with satisfactory results. The proposed NaCS/PDMDAAC/GCE provided a promising sensing platform on the detection nitrite in diverse real samples at the sub-micromolar level.

## 4. Materials and Methods 

### 4.1. Chemicals 

Poly(dimethyldiallylammonium chloride) (PDMDAAC) was supplied by Aldrich Co., Ltd (Shanghai, China). Cellulose sulfate sodium salt was purchased from J&K SCIENTIFIC LTD (Shanghai, China). Borax and sodium nitrite were purchased from Hunan General Chemical Reagent Works (Changsha, China). Potassium ferricyanide, potassium hexacyanoferrate, sodium dihydrogen phosphate and disodium hydrogen phosphate were all bought from Sinopharm Chemical Reagent Co., Ltd (Shanghai, China). All the chemicals were of analytical reagent grade and were used as received without any further purification. Both PDMDAAC and NaCS were also synthesized in our experiments (details can be seen in the Supporting Information). 

### 4.2. Fabrication of NaCS/PDMDAAC/GCE 

The synthesis of NaCS and PDMDAAC has been described in the Supporting Information. The infrared spectra of cellulose sodium sulfate, ^1^H NMR spectrum of DMDAAC, infrared spectra of (a) PDMDAAC & (b) DMDAAC have been also plotted in the Supporting Information (Figure S1, Figure S2 & Figure S3). GCE was polished to mirror-like surface with 0.3 μm and 0.05 μm alumina powder, rinsed twice with ethanol and distilled water, and dried under natural condition. Firstly, 3 μL PDMDAAC (7% (*w*/*v*)) solution was drop-casted on the surface of GCE, dried naturally to obtain PDMDAAC/GCE. Secondly, 3 μL NaCS (3.5% (*w*/*v*)) solution was dropped-casted to the surface of PDMDAAC/GCE, dried naturally to obtain NaCS/PDMDAAC/GCE. 

### 4.3. Electrochemical Measurements

All electrochemical experiments were performed on the CHI-760B electrochemical workstation (Shanghai Chenhua Instrument Co., Ltd., Shanghai, China). A standard three-electrode system is used for all electrochemical experiments, consisting of bare GCE or modified electrodes as the working electrode, platinum wire as the counter electrode, and an Ag/AgCl electrode (saturated potassium chloride) as the reference electrode. The electrochemical performance was measured by cyclic voltammetry (CV) and electrochemical impedance spectroscopy (EIS) in 5 × 10^–3^ mol·L^–1^ K_3_[Fe(CN)_6_] solution. The electrochemical behavior of nitrite at the NaCS/PDMDAAC/GCE was investigated by CV. The content of nitrite was detected by differential pulse voltammetry (DPV). The supporting electrolyte for all electrochemical measurements is 0.1 mol·L^–1^ Na_2_HPO_4_-NaH_2_PO_4_ buffer solution (PBS, pH 7.0, containing 0.1 M NaCl) unless otherwise stated.

### 4.4. Pretreatment of Ham Sausage Samples

The ham sausage samples were purchased from a local supermarket. 12.5 mL of borax saturation solution was added into 5 g ground ham sausage, and stirred to mix well. Then the ham sausage sample was washed into 50 mL volumetric flask by a small amount of hot water, and heated in boiling water bath for 15 min. Afterwards the volumetric flask was cooled to room temperature. Subsequently, 2.5 mL PBS solution was added into the volumetric flask, and then dilute with distilled water to 50 mL. After 30 min rest, the upper layer of fat or oil was removed, the spare liquor was extracted by suction filtration to obtain sample solution for quantitative analysis. 

## Figures and Tables

**Table 1:** A comparison on sensing performances between reported electrodes and NaCS/PDMDAAC/GCE.

Electrodes	Methods	Linear Ranges (μmol·L^–1^)	LOD (μmol·L^–1^)	References
POSS/rGO/GCE	CA	0.5–120	0.08	[45]
PNB/GCE	DPV	0.5–100	0.1	[46]
β-MnO_2_ NRs/GCE	CA	0.29–26,090	0.29	[47]
Ag/HNT/MoS_2_/CPE	CA	2–425	0.7	[48]
MWCNTs-TiN/GCE	CA	1–2000	0.0014	[49]
hemin/TNT/GCE	CA	0.6–130	0.084	[50]
NaCS/PDMDAAC/GCE	DPV	150–0.04	0.043	This work

**Table 2:** The reproducibility of NaCS/PDMDAAC/GCE for nitrite determination.

No.	Current Response (μA)	
	8.0 × 10−5 mol⋅L^–1^	8 × 10−6 mol⋅L^–1^
1	0.896	0.115
2	0.874	0.123
3	0.887	0.112
4	0.888	0.121
5	0.878	0.114
6	0.890	0.117
7	0.898	0.112
8	0.894	0.129
9	0.903	0.122
Average	0.889	0.118
SD	0.009	0.006
RSD (%)	1.05%	4.89%

**Table 3:** Determination of nitrite in ham sausage samples using the NaCS/PDMDAAC/GCE.

Samples	Content (mg/kg)	Added (mg/kg)	Founded (mg/kg)	Recovery (%)	RSD (%)
1	27.32	30	58.52	104.0	2.3
2	26.87	30	57.32	102.6	1.4
3	26.24	30	55.71	98.2	3.3

## Supplementary Materials

The following are available online.
