# Supplementary material for: Ultrasensitive Electrochemical Sensor Based on Polyelectrolyte Composite Film Decorated Glassy Carbon Electrode for Detection of Nitrite in Curing Food at Sub-Micromolar Level
Source: Molecules. 2018 Oct 9;23(10):2580. doi: 10.3390/molecules23102580 (PMC6222513; doi:10.3390/molecules23102580)
Supplement: Supplementary file 1 [file molecules-23-02580-s001.pdf]

Supporting information

Ultrasensitive Electrochemical Sensor based on Polyelectrolyte Composite Film Decorated Glassy Carbon Electrode for Detection of Nitrite in Curing Food at Sub-micromolar Level

1. Materials and methods

Chemicals: n-propanol, isopropyl alcohol, acetone, concentrated sulfuric acid, cellulose, sodium nitrite, anhydrous ethanol, ethylenediaminetetraacetic acid disodium salt, Dimethylamine, thymolphthalein, allyl chloride, sodium hydroxide and ammonium persulfate were purchased from Sinopharm Chemical Reagent Co., Ltd (Shanghai, China). All the chemicals were of analytical Reagent grade and were used as received without any further purification.

Instruments: Infrared spectra were acquired by Thermo Nicolet Corporation(Avatar 360, America), and ^{1}H NMR spectra were acquired by Bruker-Avance (400 M, Germany).

2. Systhesis and characterization of sodium cellulose sulfate (NaCS)

36 mL of n-propanol was slowly added into a beaker containing 46 mL of concentrated sulfuric acid with ice water bath, and stirred to prevent local overheating at the same time. If n-propanol was added too fast, the color of the solution will turn yellow quickly. Then let the mixture solution rest overnight to obtain an acid-alcohol mixture solution. 2.0135 g of dried cellulose was weighed and then added to a three-necked flask containing an acid-alcohol mixture solution, stirred and reacted in an ice water bath for 16 h. After the reaction is completed, the reaction solution is vacuum filtered, repeated washed with isopropyl alcohol three times. Subsequently, the as-obtained precipitate dissolved into 50 mL of cold deionized water, afterwards 10% sodium hydroxide solution is added dropwise to adjust pH to alkaline, and the filtrate were collected by vacuum filtration. Finally, the equal volume of ethanol was added to the as-obtained filtrate, white viscous sodium cellulose sulfate solid was precipitated out, squeezed and vacuum-dried overnight to obtained sodium cellulose sulfate.

The sodium cellulose sulfate solid was dried in a vacuum and then ground with potassium bromide, and the infrared spectrum was measured by an infrared spectrometer as shown in Fig. S1.

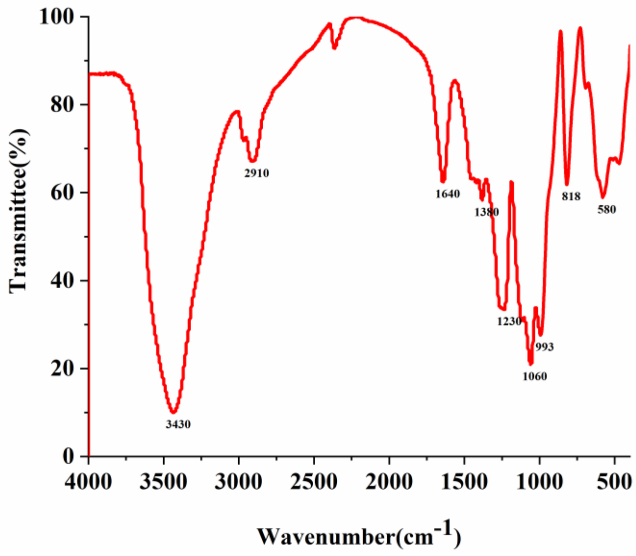

Fig.S1 Infrared spectra of cellulose sodium sulfate

In the infrared spectrum of sodium cellulose sulfate, it can be seen that the stretching vibration absorption peak of C-O-H at 2912 cm^{-1} and the stretching vibration absorption peak of carbon-hydrogen bond at 3435 cm^{-1}, 1238 cm^{-1} It is the peak of the stretching vibration absorption of S=O and -$COSO_3$, and there is a characteristic absorption peak of sulfate in the

vicinity of 800 cm^{-1}. The spectra indicate that sodium cellulose sulfate is successfully synthesized.

3. Preparation and characterization of poly(dimethyldiallylammonium chloride) (PDMDAAC)

3.1 Preparation of dimethyldiallylammonium chloride (DMDAAC)

Dimethylamine was added into a three-necked flask equipped with a reflux condenser, and stirred in an ice water bath. With thymolphthalein as indicator, the equimolar sodium hydroxide and allyl chloride were alternately added dropwise, keeping the color of indictor not change. Afterwards the mixture was stirred for 1.5 h, and then two phases were separated by a separating funnel. The upper oil phase was taken out to obtain dimethyl allyl tertiary amine (DMAA), and then sodium hydroxide solid was added into DMAA. After 2.5 h rest, an equal volume of acetone and 20% (V/V) distilled water were added separately. The allyl chloride was added dropwise, and the temperature was raised to 45 °C for 4 h. After the reaction is finished, washed repeatedly with acetone 2 – 3 times to obtain dimethyl diallyl ammonium chloride (DMDAAC).

3.2 Preparation of poly(dimethyldiallylammonium chloride) (PDMDAAC)

DMDAAC (w = 65%) was added into a four-necked flask equipped with a reflux condenser. Then, ammonium persulphate (APS) and ethylenediaminetetraacetic acid disodium salt (EDTA-2Na) were added as initiator and additive (m(APS) : m(DMDAAC) = 0.35%, m(APS) : m(DMDAAC) = 0.0035%), respectively. The temperature remains below 40 ℃, and then nitrogen gas was bubbled into the flask for 20 min to drive out the air. Afterwards, the reaction reacted at 45 ℃ for 2 h, 55 ℃ for 3 h, and 67 ℃ for 2 h. Finally, appropriate amount of acetone was added to dissolve the unreacted monomer, and then removed with the helping of the rotary evaporation apparatus.

3.3 Characterization

3.3.1 Nuclear magnetic resonance spectroscopy for DMDAAC

The dried monomer sample (DMDAAC) is dissolved in $CDCl_3$ solvent, and the sample is subjected to hydrogen spectrum measurement. The ^1H NMR spectrum of DMDAAC is shown in Fig. S2. The chemical shift values are as follows: ^1H NMR（$CDCl_3$, 400 Hz, ppm）δ: 3.32 (m, 6H, $-CH_3$), 4.50~4.52 (m, 4H, $-CH_2-$), 5.74 ~ 5.84 (m, 4H, $=CH_2$), 5.96 ~ 5.98 (m, 2H, -CH=), confirming the structure of DMDAAC, though there exists an impurity shift peak.

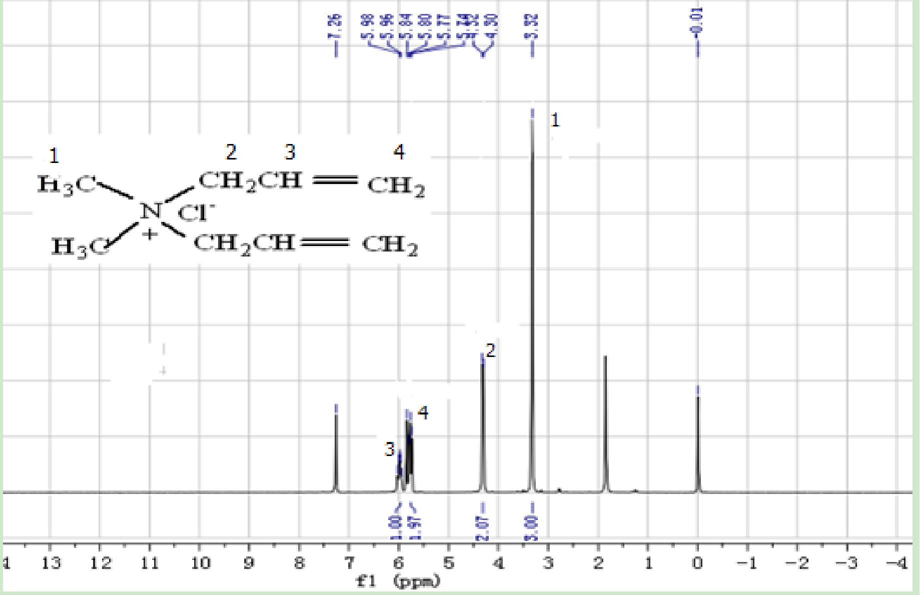

Fig.S2 ^1H NMR spectrum of DMDAAC

3.3.2 Infrared spectra for DMDAAC and PDMDAAC

After DMDAAC and PDMDAAC were dried in a vacuum, they were ground with potassium bromide, tableted, and their infrared spectra were measured by an infrared spectrometer.

The infrared spectrum is shown in Fig. S3, where the curve a and b are the infrared spectra of PDMDAAC and DMDAAC, respectively. As can be seen from Fig.3, in the curve b, 3022 cm-1 is a stretching vibration peak of a carbon-hydrogen bond from the double bond (=CH_2), 2970 cm^{-1} is a peak of stretching vibration caused by the methyl/methylene group, and 1642 cm^{-1} is a stretching vibration absorption peak of a carbon-carbon double bond. 1458 cm^{-1} is the in-plane bending vibration absorption peak at the ortho position of the N atom, while 1000 cm^{-1} and 883 cm^{-1} are out-of-plane bending vibration absorption peaks caused by the carbon-hydrogen bond and the double bond, respectively. In the curve a, it can be seen that the typical infrared absorption peaks at 3093 cm^{-1}, 3011 cm^{-1}, 1643 cm^{-1} and 853~876 cm^{-1}, have been significantly weakened or disappeared, indicating DMDAAC has been polymerized into PDMDAAC.

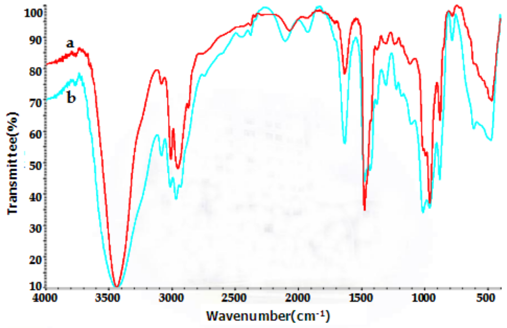

Fig.S3 Infrared spectra of (a)PDMDAAC; (b)DMDAAC
